# Using the bootstrap to establish statistical significance for relative validity comparisons among patient-reported outcome measures

**DOI:** 10.1186/1477-7525-11-89

**Published:** 2013-05-31

**Authors:** Nina Deng, Jeroan J Allison, Hua Julia Fang, Arlene S Ash, John E Ware

**Affiliations:** 1 Department of Quantitative Health Sciences, University of Massachusetts Medical School, Worcester, MA, 01655, USA; 2 John Ware Research Group, Incorporated, One Innovation Drive, Suite 400, Worcester, MA, 01605, USA

**Keywords:** Bootstrap, Relative validity, Analysis of variance (ANOVA), Confidence interval, Patient-reported outcome (PRO) measure, Chronic kidney disease (CKD)

## Abstract

**Background:**

Relative validity (RV), a ratio of ANOVA F-statistics, is often used to compare the validity of patient-reported outcome (PRO) measures. We used the bootstrap to establish the statistical significance of the RV and to identify key factors affecting its significance.

**Methods:**

Based on responses from 453 chronic kidney disease (CKD) patients to 16 CKD-specific and generic PRO measures, RVs were computed to determine how well each measure discriminated across clinically-defined groups of patients compared to the most discriminating (reference) measure. Statistical significance of RV was quantified by the 95% bootstrap confidence interval. Simulations examined the effects of sample size, denominator F-statistic, correlation between comparator and reference measures, and number of bootstrap replicates.

**Results:**

The statistical significance of the RV increased as the magnitude of denominator F-statistic increased or as the correlation between comparator and reference measures increased. A denominator F-statistic of 57 conveyed sufficient power (80%) to detect an RV of 0.6 for two measures correlated at r = 0.7. Larger denominator F-statistics or higher correlations provided greater power. Larger sample size with a fixed denominator F-statistic or more bootstrap replicates (beyond 500) had minimal impact.

**Conclusions:**

The bootstrap is valuable for establishing the statistical significance of RV estimates. A reasonably large denominator F-statistic (F > 57) is required for adequate power when using the RV to compare the validity of measures with small or moderate correlations (r < 0.7). Substantially greater power can be achieved when comparing measures of a very high correlation (r > 0.9).

## Introduction

There has been an increasingly widespread application of patient-reported outcome (PRO) measures in assessing the outcomes of health-related quality of life. Along with the noteworthy improvements in measurement theory, advances in data capture and processing technologies, and various approaches to generic and disease-specific measures, there are more available choices among PRO measurement tools than ever before. Relative Validity (RV), also referred to as relative precision or relative efficiency [1]–[3], provides an appropriate quantitative index to compare the validity of PRO measures under the conditions in which such measures are typically used. As such, the RV compares two PRO measures on their ability to discriminate patients across disease severity levels and on their ability to detect longitudinal change [4]–[6]. Complementary to other psychometric properties such as reliability and respondent burden, the RV is used frequently in literature providing important validity information of PRO measures.

A noteworthy limitation of the RV, which we address in this study, is the absence of a basis for establishing its statistical significance and an understanding of the factors affecting that significance. The common practice is to compute the RV and simply conclude that the comparator measure has more (or less) discriminating power or responsiveness than the reference measure if the RV is greater (or less) than 1. However, an RV may differ from the null value of 1 because of the random error in the absence of “true” differences among the measures being compared. Therefore, establishing the statistical significance of the RV is necessary for identifying “true” differences in validity between PRO measures.

In spite of this need, the statistical significance of the RV is typically not discussed, likely because its underlying probability distribution is not easily derived analytically. The bootstrap, a well-known statistical technique for estimating the confidence interval based on an empirical distribution without assuming a probability distribution, offers a promising solution [7]–[10]. This technique, however, has not yet been widely applied to the RV. Therefore, we evaluated the bootstrap as a technique for statistically testing the RV and, furthermore, used simulations to investigate factors that may affect the bootstrap confidence intervals of the RV under different conditions.

## Methods

### Description of the data

Secondary data analyses were conducted using the responses of 453 chronic kidney disease (CKD) patients to sixteen CKD-specific and generic PRO measures. The 16 measures included (a) three widely-used CKD-specific legacy scales: the Kidney Disease Quality of Life (KDQOL) Burden, Symptoms, and Effects scales [11]; (b) eight generic profile scales that are widely used in CKD: the Medical Outcomes Study Short-Form 12 (SF-12) with Physical Functioning (PF), Role Physical (RP), Bodily Pain (BP), General Health (GH), Vitality (VT), Social Functioning (SF), Role Emotional (RE), and Mental Health (MH); (c) two generic summary scales included in the SF-12: Physical Component Summary (PCS) and Mental Component Summary (MCS) [12]; and (d) three varying-in-length forms of the newly developed Quality-of-life Disease Impact Scale for CKD (QDIS-CKD) [13,14]: the original 34-item form – Static-34, a shorter 6-item form – Static-6, and a computer adaptive testing (CAT) form with five dynamic items – CAT-5. The 16 measures were chosen to allow comparison of widely-used generic and CKD-specific measures and to compare new measures with the legacy measures. External clinically-defined disease states were used to classify the patients into three ANOVA groups: dialysis (n = 206), pre-dialysis stage 3-5 (n = 113), and transplant (n = 134) [15]–[17]. The study was approved by the New England Institutional Review Board (NEIRB 06-058). Patients were fully informed and consent was obtained.

### Relative validity

The relative validity (RV) is defined as a ratio of ANOVA F-statistics, with the F-statistic of the comparator measure taken as the numerator and the F-statistic of the reference measure taken as the denominator. An RV greater than 1 indicates that the comparator measure has greater discriminating power or responsiveness than the reference measure, and vice versa. This approach for validating PRO measures is also called the “known-groups method” [18] because the F-statistic is obtained by comparing groups known to differ based on external criteria, e.g., clinically-defined diagnosis or severity. We prefer the term relative validity because separation between known groups as measured by the F-statistic is the essence of validity. In addition to comparing different PRO measures, the RV is also widely used for comparing different scoring methods for the same PRO measure, e.g., the classical summed score versus the score based on modern psychometric models such as the item response theory (IRT) models [19]–[23].

The RVs were computed for the 16 CKD-specific and generic PRO measures. Given that there is no gold-standard measure available, the QDIS-CKD CAT-5, the measure with the largest F-statistic, was chosen as the reference measure. The RVs were computed to independently evaluate each measure’s relative validity in discriminating among patients across the three clinical groups compared to the reference measure. Summary statistics for the data, including the group sample sizes, means, standard deviations, F-statistics, and RVs, are displayed in Table 1. It is of note that two PRO measures (SF-12 MCS and MH) had small and non-significant F-statistics and could not effectively discriminate across the clinical groups. Therefore, we did not calculate their RVs nor analyze them further.

**Table 1:** ANOVA-based F-statistic and relative validity for CKD-specific and generic PRO measures across clinically-defined groups (N = 453)

**PRO measure**	**Dialysis**			**Pre-dialysis stage 3-5**			**Transplant**			***r***^***b ***^**(total)**	**F-statistic**	**RV**	**95% CI**^**c**^
	**(n = 206)**			**(n = 113)**			**(n = 134)**						
	**Mean**	**(SD)**	***r***^**a**^	**Mean**	**(SD)**	***r***^**a**^	**Mean**	**(SD)**	***r***^**a**^				
*CKD-specific*													
QDIS-CKD													
CAT-5	39.83	(22.17)	1	16.19	(21.51)	1	19.25	(21.63)	1	1	57.43^**^	1	-
Static-6	39.18	(22.86)	0.91	16.86	(21.84)	0.96	19.60	(21.29)	0.93	0.94	50.15^**^	0.87	(0.72-1.03)
Static-34	35.93	(21.23)	0.93	14.90	(20.05)	0.96	18.71	(20.52)	0.95	0.95	48.01^**^	0.84	(0.71-0.97)
KDQOL													
Burden	48.83	(26.81)	-0.60	76.62	(24.76)	-0.73	68.21	(28.90)	-0.77	-0.74	44.46^**^	0.77	(0.53-1.09)
Symptoms	71.95	(16.23)	-0.57	80.58	(15.80)	-0.65	80.03	(15.96)	-0.66	-0.65	15.11^**^	0.26	(0.13-0.44)
Effects	63.41	(21.92)	-0.54	84.38	(17.59)	-0.79	77.86	(20.18)	-0.73	-0.71	43.95^**^	0.77	(0.52-1.10)
*Generic*													
SF-12													
PF	37.06	(10.75)	-0.52	45.38	(11.12)	-0.57	44.88	(10.69)	-0.65	-0.63	31.12^**^	0.54	(0.32-0.85)
RP	38.00	(9.41)	-0.65	45.12	(9.78)	-0.61	45.83	(9.91)	-0.63	-0.69	34.12^**^	0.59	(0.38-0.89)
BP	43.19	(11.67)	-0.47	46.71	(11.27)	-0.53	47.10	(11.66)	-0.49	-0.50	5.84^**^	0.10	(0.02-0.22)
GH	39.08	(11.19)	-0.47	41.99	(10.11)	-0.51	43.71	(10.93)	-0.56	-0.52	7.79^**^	0.14	(0.04-0.28)
VT	45.72	(9.25)	-0.44	46.40	(10.15)	-0.46	48.35	(9.93)	-0.48	-0.44	3.04^*^	0.05	(0.00-0.15)
SF	42.75	(11.79)	-0.65	47.81	(11.25)	-0.61	47.83	(10.78)	-0.60	-0.64	11.02^**^	0.19	(0.07-0.34)
RE	44.59	(11.64)	-0.52	48.39	(10.05)	-0.49	48.39	(9.76)	-0.42	-0.51	7.01^**^	0.12	(0.03-0.25)
PCS	36.60	(10.29)	-0.54	43.49	(10.37)	-0.56	44.08	(10.72)	-0.66	-0.64	26.61^**^	0.46	(0.27-0.74)
MCS^d^	49.74	(10.38)	-0.49	50.42	(9.57)	-0.45	50.55	(9.94)	-0.32	-0.40	0.32	-	-
MH^d^	49.85	(10.39)	-0.43	50.71	(10.25)	-0.49	50.31	(9.95)	-0.33	-0.38	0.26	-	-

^*^Significant at the 0.05 level ^**^ Significant at the 0.01 level.

*Abbreviations: ANOVA* analysis of variance, *RV* relative validity, *CKD* chronic kidney disease, *PRO* patient-reported outcome, *QDIS-CKD* quality-of-life disease impact scale for chronic kidney disease, *KDQOL* kidney disease quality-of-life, *SF-12* Short Form 12, *PF* physical functioning, *RP* role physical, *BP* bodily pain, *GH* general health, *VT* vitality, *SF* social functioning, *RE* role emotional, *PCS* physical component summary, *MCS* mental component summary, *MH* mental health.

^a^*r* refers to the correlation of scores between each comparison measure and the reference measure (QDIS-CKD CAT-5) within each clinical group.

^b^*r*(total) refers to the correlation of scores between each comparison measure and the reference measure (QDIS-CKD CAT-5) for the total group (N = 453).

^c^ The 95% confidence interval of the RV was derived from the original data using the bootstrap BCa interval.

^d^ The F-statistics for SF-12 MCS and MH are small and non-significant ( p-values of 0.73 and 0.77 separately), therefore their RVs were not computed and excluded from significance test.

### Bootstrap technique

We used bootstrap technique to estimate the standard error (SE) and 95% confidence interval (CI) for the RVs [20]–[26]. The bootstrap is a statistical technique for estimating the accuracy of an estimator and is available in many commonly used statistical software packages. Under the assumption that the empirical distribution of the observed data well represents the true population distribution, the bootstrap technique randomly re-samples with replacement the empirical distribution with the sample size equal to the empirical sample size. This technique thus creates multiple “bootstrap replicate” samples, and then computes the RV for each replicate to approximate the sampling distribution of the RV. The standard deviation of RVs from the bootstrap replicates becomes the standard error of the RV estimate, indicating the size of uncertainty (error) in the point estimate of the RV. The 2.5^th^ and 97.5^th^ percentiles of the bootstrap distribution of the RV provide the basis for the 95% confidence interval (CI), which is a range designed to capture with 95% probability the “true” value of RV. Statistical significance of the RV is implied by 95% confidence intervals that exclude the null value of 1.

There are several types of bootstrap confidence intervals available, e.g., the normal, percentile, and bias-corrected intervals, etc. The bias-corrected and accelerated (BCa) interval is generally considered superior to other methods and therefore was chosen for this study [27]. The BCa interval computes an *adjusted* percentile confidence interval that accounts for the possible bias of the bootstrap distribution introduced by the re-sampling process and the variable variance of the bootstrap replicates [28]. In addition, under the circumstances that the bootstrap distributions are potentially biased and skewed, the relationship between the bootstrap standard error and the BCa interval is not quite straightforward; therefore, we reported both the bootstrap standard error and the BCa interval as the complementary information to evaluate the accuracy of RV estimates.

### Simulation studies

It is rather intuitive that an RV of 0.3 would more likely be detected as significantly different from the null value of 1 than an RV of 0.6. However, we lack an understanding of the conditions which might cause a given RV to be statistically significant in one study but not in another. Therefore, simulations were conducted to evaluate the potential effects of various factors on the bootstrap results of the RV. Four important factors were manipulated and investigated : (1) sample size (N = 100, 200, 300, 453, 600, 1000, and 2000), (2) magnitude of the F-statistic for the reference measure (F = 12.6, 25.4, 38.0, 57.4, 76.1, 126.8, and 253.6), (3) magnitude of correlation between the comparator and the reference measure ( *r* = 0, 0.3, 0.5, 0.7, 0.9, and 0.95), and (4) number of bootstrap replicates (B = 500, 1000, and 2000). Factors not varied were retained as found in the original sample. Each study is described in more detail below.

#### Sample size

We initially suspected that the sample size would play a prominent role in determining the bootstrap confidence interval of the RV. Seven sample sizes were examined: N = 100, 200, 300, 453, 600, 1000 and 2000, where 453 was the original data sample size. Group means, standard deviations, and correlations between the comparator and reference measure were retained as in the original dataset, as well as the proportion of patients in the three clinical groups (45%, 25% and 30% respectively). It is worthy of note that for a given data set, by definition, the F-statistic increases as the sample size increases with constant group means and standard deviations. For example, the F-statistics of the reference measure (QDIS-CKD CAT-5) were 12.6, 25.4, 38.0, 57.4, 76.1, 126.8, and 253.6 for the seven proposed sample sizes, respectively.

The situation does arise in which we want to assess the effects of sample size independent of the F-statistic. For example, when we evaluate the RVs computed in two data sets, a larger sample size does not necessarily translate into a larger F-statistic because of differences in group means and standard deviations. Yet, we would still be interested in knowing whether the larger sample size produces a more precise RV estimate. For this reason, we used a second design to assess the effects of sample size while holding the F-statistic constant. To implement this approach, we let N^*^ denote the desired sample size in the simulated data, N denote the original sample size, and T = N / N^*^. By multiplying the group standard deviation by 1/√T and keeping the group mean constant, the F-statistic remained fixed at the values observed in the original data (see Table 1) across the different sample sizes. In particular, the F-statistic of reference measure (QDIS-CKD CAT-5) was fixed at 57.4, as found in the original data. Because the F-statistic remained fixed for all PRO measures, the RVs were held constant across the simulated sample size conditions.

#### F-statistic of reference measure (denominator F-statistic)

Beyond the sample size, we suspected that the magnitude of the denominator F-statistic of the RV would play an important role in determining the statistical significance. Consider four PRO measures A, B, C and D with F-statistics of 60, 100, 6, and 10, respectively. We suspected that the difference between measures A and B would be more significant than the difference between measures C and D, although both comparisons yield RV = 0.6. The hypothesis was that, given equal RVs, a greater F-statistic for the reference measure would be associated with a smaller standard error and a greater power.

To test this hypothesis, we simulated data with different F-statistics but a fixed sample size, so that the effect of the magnitude of F-statistic could be examined separately. Similar to the design described above, we let F^*^ denote the desired F-statistic in the simulated data, F denote the F-statistic observed in the original data, and T^*^ = F^*^ / F. By multiplying the group standard deviation by 1/√T^*^ and keeping the sample size and group mean constant, the F-statistics changed by a factor of T^*^. To promote convenient comparisons, data were generated with F-statistics corresponding to those obtained in the first design of the sample size condition (e.g., the F-statistics of reference measure were simulated at 12.6, 25.4, 38.0, 57.4, 76.1, 126.8, and 253.6, respectively). In agreement with the original data, the total sample size was fixed at 453. Note that because the F-statistic changed by the same factor of T^*^ for all PRO measures, the RVs were again held constant across the simulated conditions of F-statistics.

#### Correlation between comparator and reference measures

We noted moderate to high correlations among the PRO measures in the study. Furthermore, it seemed appropriate to assume moderate correlations between measures developed for very similar concepts, and even higher correlations between measures sharing common questions (e.g., short and long forms). Specifically, the alternative forms of QDIS-CKD (Static-6 and Static-34) were more highly correlated with the reference measure of QDIS-CKD CAT-5 than the scales of KDQOL or SF-12 (Table 1). Additionally, we observed that the RV for QDIS-CKD Static-34 (RV = 0.84) was statistically significant while the RVs for KDQOL Burden and for KDQOL Effects (both equal to 0.77) were not, despite the latter being further from the null value of 1. This suggested that a greater correlation between the comparator and the reference measure may lead to greater precision in the RV estimate.

To test this hypothesized effect of correlation, data were generated with a wide range of correlations between the comparator and the reference measures: *r* = 0, 0.3, 0.5, 0.7, 0.9 and 0.95. The within-group correlations were assumed equal across the three severity groups. Group means, standard deviations, and sample sizes were maintained constant as in the original data. Again, the RVs for all PRO measures were held constant across the different conditions of correlations.

#### Number of bootstrap replicates

How many bootstrap replicates are needed in practice to compute stable 95% confidence intervals for the RV statistic? Efron and Tibshirani [27] suggested 200 for calculating the bootstrap standard error but 1000 or more for computing the bootstrap confidence interval. Some researchers have suggested even larger numbers [9]. Because the BCa interval is computationally intensive, minimizing the number of bootstrap replicates might convey practical benefits. Three batches of bootstrap replicates (B = 500, 1000, 2000) were tested. Group sample sizes, means, standard deviations and correlations were all kept constant as in the original data.

### Simulation steps

The simulation was programmed in the language R. The package “boot” was used for bootstrapping, including computing bootstrap standard errors and BCa confidence intervals [29]–[31]. Separate simulations were conducted for each condition of sample sizes, denominator F-statistics, and correlations between the comparator and reference measures. In each simulation, factors not varied were fixed at the observed data as shown in Table 1. For each simulation, the steps below were followed.

1. Generate response data from a bivariate normal distribution for each ANOVA group and for each pair of comparator and reference measures. Using the underlying assumption for ANOVA and F-statistic, we thus simulated the data from the normal distribution.

2. Repeat Step 1 for 100 times to establish 100 simulation datasets for each study condition.

3. For each simulation dataset, calculate the bootstrap standard error and the 95% bootstrap confidence interval for the RVs.

4. Calculate the average bootstrap standard error, the average 95% bootstrap confidence interval, and the proportion of significant RVs (the “power*”*) across the 100 simulation datasets.

## Results

### Bootstrap standard error

**Figure 1:**
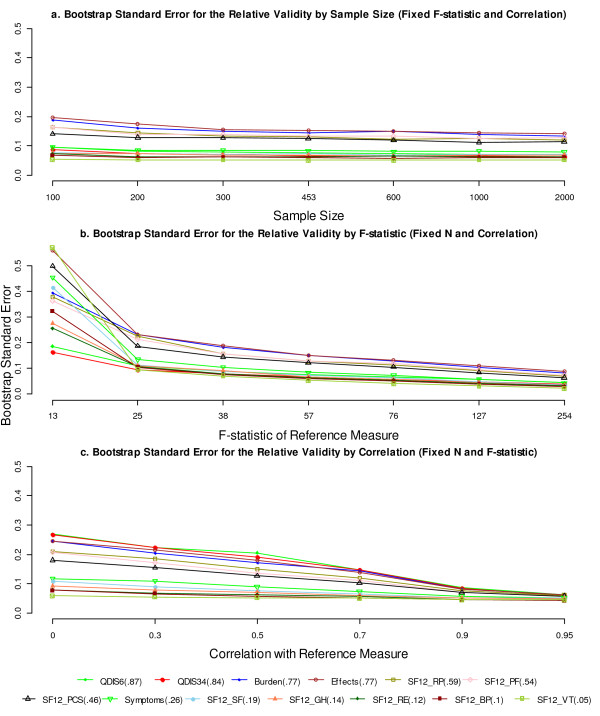
**Bootstrap standard error of the RV by sample size, denominator F-statistic, and correlation between measures.** Factors not varied in simulation were held constant as in Table 1. The RVs were held constant in simulations and are parenthesized next to the measures in the legend. *Abbreviations:* RV, relative validity; QDIS, quality-of-life disease impact scale; KDQOL, kidney disease quality-of-life; SF-12, Short Form 12; PF, physical functioning; RP, role physical; BP, bodily pain; GH, general health; VT, vitality; SF, social functioning; RE, role emotional; PCS, physical component summary.

Figure 1 displays how the bootstrap standard error of RVs varied independently by sample size, denominator F-statistic, and correlation between the comparator and reference measures. The bootstrap standard error changed little when the sample size increased with a fixed F-statistic (Figure 1a). By contrast, the standard error decreased substantially when the F-statistic increased with a fixed sample size (Figure 1b). This decrease was most substantial when the F-statistic of the reference measure was between 13 and 25. Finally, the bootstrap standard error decreased steadily as the correlation between the comparator and the reference measures increased (Figure 1c). The decrease was the most substantial at the correlation of 0.9 or above. It is noteworthy that when both the F-statistic and the sample size increased (the first design in the sample size conditions), the standard error of the RV decreased in a highly similar pattern as presented in Figure 1b. In short, the plots suggest that both the magnitude of the denominator F-statistic and the correlation between the comparator and reference measures have a substantial effect on the precision of the RV estimate, while the sample size effect was almost entirely conveyed through its influence on the magnitude of the denominator F-statistic.

### Bootstrap confidence interval and power

**Figure 2:**
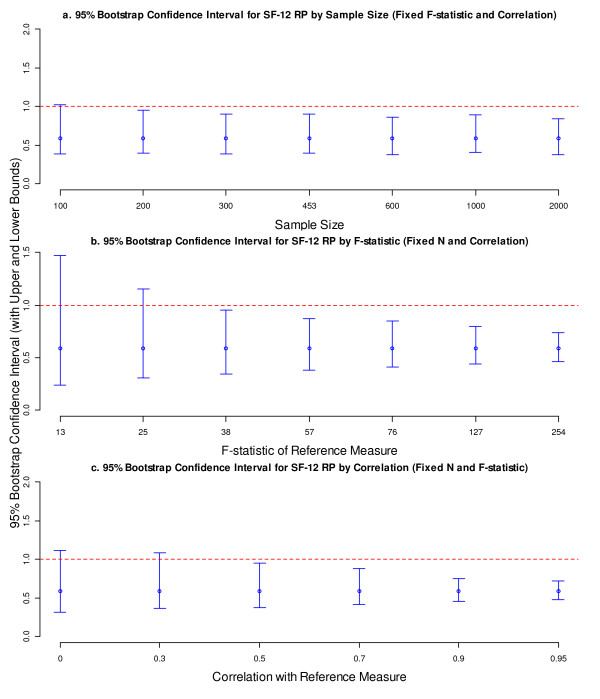
**Bootstrap confidence interval for the RV of SF-12 RP by sample size, denominator F-statistic, and correlation.** The vertical bars present the confidence intervals with upper and lower bounds. The dots on the vertical bars represent the point estimates of RVs. *Abbreviations:* RV, relative validity; SF-12, Short Form 12; RP, role physical.

Consistent with results of the bootstrap standard error, we found little change in the bootstrap confidence interval when the sample size changed independently of the F-statistics, although there was a noticeable change the other way around. Using the PRO measure of SF-12 RP (RV = 0.59) as a specific example, Figure 2 displays the average 95% bootstrap confidence intervals (represented by the vertical bars) under various simulation conditions. In general, the confidence interval was relatively insensitive to varied sample size with a fixed F-statistic (Figure 2a), but became increasingly narrower as either the denominator F-statistic or the correlation between the comparator and reference measures increased (Figures 2b and 2c). More specifically, the confidence interval became significant – by excluding 1 – when either the denominator F-statistic or the correlation became greater.

**Figure 3:**
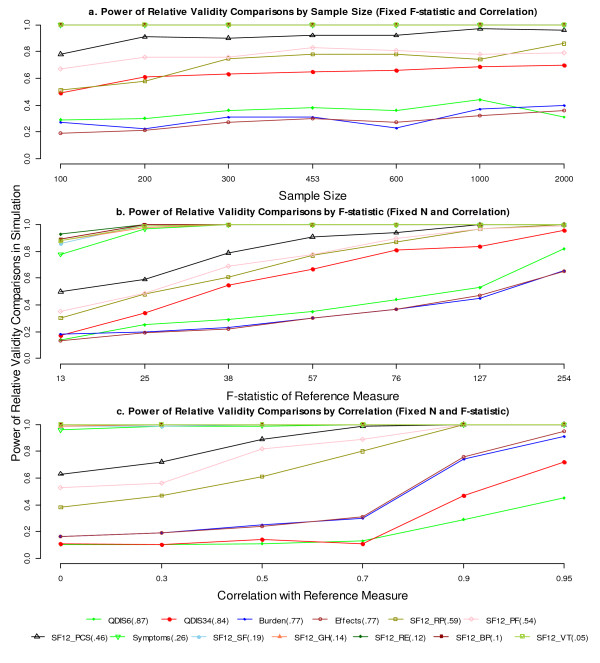
**Power of the RV by sample size, denominator F-statistic, and correlation between measures.** Power was calculated as the probability of obtaining a statistically significant RV from simulations. Factors not varied in simulation were held constant as in Table 1. The RVs were held constant in simulations and are parenthesized next to the measures in the legend. *Abbreviations:* RV, relative validity; QDIS, quality-of-life disease impact scale; KDQOL, kidney disease quality-of-life; SF-12, Short Form 12; PF, physical functioning; RP, role physical; BP, bodily pain; GH, general health; VT, vitality; SF, social functioning; RE, role emotional; PCS, physical component summary.

Power was calculated as the proportion of significant RVs across the simulation replications. Likewise, the power increased as the denominator F-statistic or the correlation between the comparator and reference measure increased (Figures 3). Specifically, the power for detecting low RVs (RVs = 0.05 - 0.26) remained consistently high across varied conditions. Power for moderate RVs (RVs = 0.46, 0.54, and 0.59) increased steadily as either the denominator F-statistic or the correlation increased. For example, the power for RV = 0.59 (SF-12 RP) reached 80% when the denominator F-statistic was 57 with a correlation of 0.7. Power for detecting moderately high RVs (RVs = 0.77, 0.84, and 0.87) increased fairly slowly as the denominator F-statistic increased, but much more quickly when the correlation reached 0.9 or above.

### Simulated distribution of RV estimates

**Figure 4:**
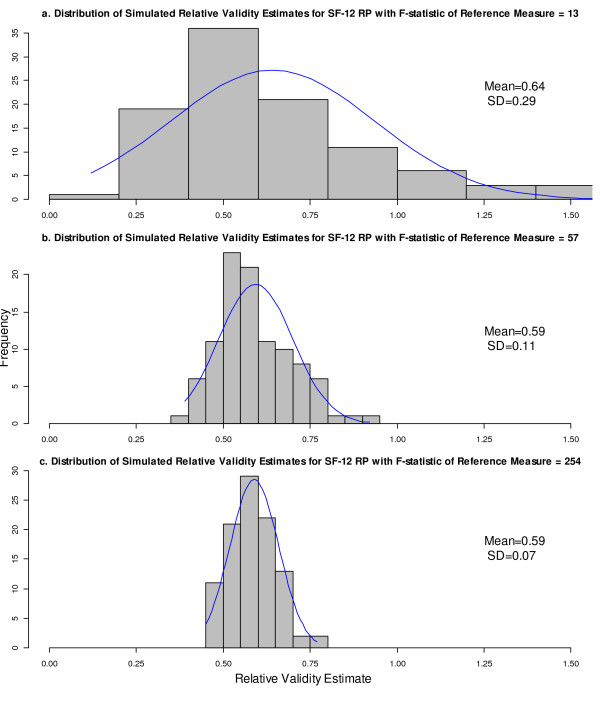
**Histogram of simulated RV estimates for SF-12 RP by denominator F-statistic.** The SF-12 RP had RV = 0.59. The sample size and correlation with the reference measure were fixed at the values observed in the original data. *Abbreviations:* RV, relative validity; SF-12, Short Form 12; RP, role physical.

Because the bootstrap is used to approximate the “true” RV sampling distribution, we expected the RV sampling distribution obtained by simulation to be consistent with the bootstrap distribution. Therefore, the sampling distributions of the RV from simulation were plotted as a validity check of the bootstrap results. Again, the SF-12 RP was illustrated as an example. The histograms of RV estimates for SF-12 RP (RV = 0.59) were plotted with the fitted normal curves when the denominator F-statistics were at 13, 57, and 254, respectively (Figure 4). In short, we found that as the denominator F-statistic increased: (1) the standard deviation of the sampling distributions decreased, consistent with changes in the bootstrap standard error, and (2) the mean of sampling distributions was closer to the “true” RV of 0.59, suggesting smaller bias and skew, again showing consistency with the bootstrap results.

### Number of bootstrap replicates

**Figure 5:**
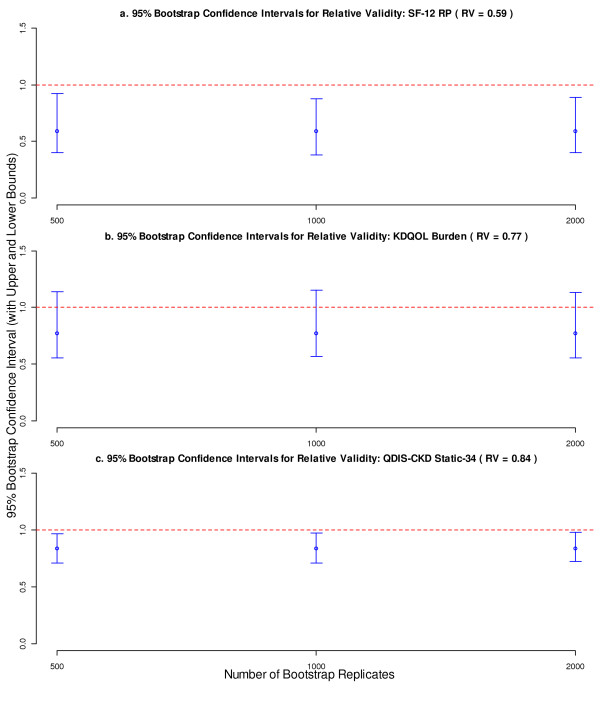
**Bootstrap confidence intervals for RVs of SF-12 RP, KDQOL Burden, and QDIS-CKD Static-34 by number of bootstrap replicates.** The sample size, denominator F-statistic, and correlation with reference measure were fixed as in Table 1. The vertical bars present the confidence intervals with upper and lower bounds. The dots on the vertical bars represent the point estimates of RVs. *Abbreviations:* RV, relative validity; SF-12, Short Form 12; RP, role physical; KDQOL, kidney disease quality-of-life; QDIS, quality-of-life disease impact scale; CKD, chronic kidney disease.

Finally, the bootstrap standard error and 95% CI were compared for different numbers of bootstrap replicates. In general, the number of bootstrap replicates, ranging from 500 to 2000, had little effect on either bootstrap standard error or confidence interval. As an example, the bootstrap 95% CIs of three selected PRO measures (RVs = 0.59, 0.77 and 0.84, respectively) are presented as a function of the number of bootstrap replicates (Figure 5). It appears that for this data set, 500 bootstrap replicates are adequate to compute stable bootstrap confidence intervals for the RV.

## Conclusions

This study demonstrated that the RV, which is often used to compare the validity of alternative PRO measures, may be statistically tested via the bootstrap confidence interval. Simulations identified two key factors affecting whether a given RV represents a statistically significant finding: the magnitude of the denominator F-statistic (the F-statistic for the reference measure), and the correlation between the comparator and the reference measure. Although, we found that a lager sample size with a fixed denominator F-statistic had limited impact on the precision of the RV estimate, it is noteworthy that for a given data set (assuming constant group means and standard deviations), increasing the sample size would produce greater power by naturally increasing the denominator F-statistic. However, we need to be careful when evaluating RVs calculated in different datasets, where a larger data set may not necessarily have a larger denominator F-statistic, and thus may not provide greater power.

More specifically, our study suggested that a denominator F-statistic as low as 13 had very limited power to detect meaningful differences in the RVs. A denominator F-statistic as large as 57 conveyed sufficient power (80%) to detect a moderate RV of 0.6, given that the measures were correlated at r = 0.7. Furthermore, we found that a greater correlation between the comparator and reference measures with the same denominator F-statistic provided greater power to detect the differences in the RVs. Based on the reduction in the bootstrap standard error (Figure 1.c) and the increase in power (Figure 3.c), we classified the correlation as small (r ≤ 0.5), moderate (0.5 < r ≤ 0.7), high (0.7 < r ≤ 0.9), or very high (r > 0.9). We also note that a very high correlation is associated with substantial gain in precision and power of the RV estimate.

## Discussion

This study has important implications for studies using the RV to compare the validity of PRO measures. First, this work demonstrates the importance of calculating the confidence interval and determining statistical significance of the RV when comparing the validity of PRO measures. Second, our findings suggest that RVs of equal size but calculated under different comparison conditions have distinct statistical implications and should be interpreted differently. A review of about 40 articles published in three relevant journals (*Journal of Clinical Epidemiology*, *Medical Care*, and *Quality of Life Research*) between 1990 and 2012 revealed that the circumstances under which the RV was computed varied widely. The sample size per ANOVA group ranged broadly from 42 to near 4000 [32,33], the F-statistic of reference measure ranged widely from less than 4 to over 400 [12,24], and the correlation between the comparator and reference measures was rarely reported. We suspect that most studies, without constructing a confidence interval for the RV estimate, over-interpreted the observed differences in the RVs with small denominator F-statistics, ignoring the possibility of falsely rejecting the null hypothesis of no difference when only chance was in operation. On the other hand, “small” but possibly meaningful and statistically significant differences may have been overlooked.

This work also has important implication for designing future studies using the RV. In planning for power calculations in such studies, we suggest that researchers begin with reasonable estimates of the correlation between the comparator and reference measures along with the ANOVA group means and standard deviations. Armed with these estimates, the investigators will better understand how to control the sample size to achieve a desired magnitude of denominator F-statistic for sufficient power. The effect of correlation between measures on the RV is important given that there is an increasing interest in developing more “efficient” forms from the same item bank [34]. Thus, it becomes very realistic to assume that the PRO measures with the same questions but varying in length are very highly correlated (r > 0.9) for the same group of respondents, as the alternative forms of QDIS-CKD (CAT-5, Static-6, and Static-34) presented in our current study. Furthermore, it seems reasonable to assume at least moderate correlations (r > 0.5) for measures assessing similar concepts but having different questions, such as the different CKD-specific measures, or the CKD-specific and generic measures with common domains. Our findings also suggest lower correlations (r < 0.5) for measures of distinct domains, such as the physical and mental health.

All confidence intervals in this study were based on the bias-corrected and accelerated (BCa) bootstrap method. Generally, there is wide consensus that this method is preferred over other methods [27]. However, there are a few caveats. First, if the acceleration parameter is small (< 0.025), then some simulations suggest that coverage of the BCa interval may be erratic. Second, if there is no bias, meaning that the bootstrap distribution is not skewed and the center of the bootstrap distribution is very close to the center of the observed distribution, bias correction may decrease the precision and unnecessarily increase the width of the BCa interval [35]. Therefore, under the circumstances of no bias and minimal acceleration, the percentile-based confidence interval may offer some advantage. However, we would urge caution because these "ideal" circumstances are not likely to be found in real studies. In fact, we found important bias and substantial acceleration factors in our bootstrap simulations.

This study has specific limitations worth consideration. First, the simulations were based on one data set of PRO measures administered to CKD patients. Nevertheless, it is expected that the findings could be generalizable to PRO measures in other conditions. That said, validations using different samples and conditions are desired. In addition, we limited the number of simulation replications to 100 for most simulation conditions. Selected comparisons were made with a much larger number of simulation replications, and similar results were found. Finally, we selected the reference measure which had the largest F-statistics and thus limited the values of RVs below the null value of 1. Nevertheless, the statistical significance of the RV should not be affected by the choice of the reference measure, and we would like to further investigate this in the future. Out next and follow-up plan is to have a more comprehensive study with additional conditions and data sets. It is hoped that such a comprehensive simulation study will provide some practical guidance with a look-up table suggesting minimum denominator F-statistics required for sufficient power to detect a range of RVs (both below and above 1) under varying circumstances (e.g., measures with different degrees of correlations).

It is noteworthy that the methodology of the RV proposed in the study is appropriate only when the assumptions of ANOVA are met. These assumptions include independent observations, normally distributed dependent variable within groups, and homogeneity of variances across groups. That stated, it is also well recognized that ANOVA is quite robust to deviations from normality and violations of homogeneous variance [36,37]. To implement this methodology, it would be ideal to have all respondents complete all measures being compared, as in our current study. However, in longer surveys this could greatly increase respondent burden. Therefore, one potential approach would be to randomize respondents to complete only selected measures. However, to achieve the randomization, the sample sizes of ANOVA groups should be approximately equal (if equal sample sizes for the measures) or proportionally the same (if unequal sample sizes for the measures) for the measures, so that their F-statistics are comparable.

Finally, when evaluating the statistical significance of the RV, it is important to recognize that a low power increases the risk of failing to detect clinically important differences, and that a very large power could convey statistical significance upon clinically trivial differences. Therefore, differences in measures should always be considered clinically, for example, by accounting for the proportions of patients misclassified using the different PRO measures.

### Consent

Written informed consent was obtained from the patient for publication of this report.

## Abbreviations

RV: Relative validity; PRO: Patient-reported outcome; CI: Confidence interval; SE: Standard error; ANOVA: Analysis of variance; CKD: Chronic kidney disease; QDIS-CKD: Quality-of-life disease impact scale for chronic kidney disease; KDQOL: Kidney disease quality-of-life; SF-12: Short Form 12; PF: Physical functioning; RP: Role physical; BP: Bodily pain; GH: General health; VT: Vitality; SF: Social functioning; RE: Role emotional; PCS: Physical component summary; MCS: Mental component summary; MH: Mental health; IRT: Item response theory

## Competing interests

The authors declare that they have no competing interests.

## Authors' contributions

ND carried out the whole study and drafted the manuscript. JJA made contributions to the research design, conceptualization, and applications of the bootstrap and simulation techniques, and provided mentoring on writing. HJF provided technical consultation on the simulation. ASA provided consultation on general statistical concepts and analyses. JEW is the principal investigator of the study from which the data was collected. JEW provided the initial study idea, participated in the research design and provided inputs and guidance throughout the study. All authors were heavily involved in drafting and editing the manuscript, and approved the final manuscript.

## References

[B1] McHorney CA Ware JE Jr Rogers W Raczek AE Lu JFR The validity and relative precision of MOS short- and long- form Health Status Scales and Dartmouth COOP Charts: Results from the Medical Outcomes Study Medical Care 1992 30 Suppl 5 MS253 MS265 1583937 10.1097/00005650-199205001-00025

[B2] Fayers MP Machin D Quality of life: The assessment, analysis and interpretation of patient-reported outcomes 2007 Chichester, England: Wiley

[B3] Luo N Johnson JA Shaw JW Coons SJ Relative efficiency of the EQ-5D, HUI2, and HUI3 index scores in measuring health burden of chronic medical conditions in a population health survey in the United States Medical Care 2009 47 53 60 10.1097/MLR.0b013e31817d92f8 19106731

[B4] Liang MH Fossel AH Larson MC Comparisons of five health status instruments for orthopedic evaluation Med Care 1990 7 632 642 2366602 10.1097/00005650-199007000-00008

[B5] Kosinski M Keller SD Ware JE Jr Hatoum HT Kong SX The SF-36 Health Survey as a generic outcome measure in clinical trials of patients with osteoarthritis and rheumatoid arthritis: Relative validity of scales in relation to clinical measures of arthritis severity Medical Care 1999 37 Suppl 5 MS23 MS39 10335741 10.1097/00005650-199905001-00003

[B6] Werneke M Hart DL Discriminant validity and relative precision for classifying patients with nonspecific neck and back pain by anatomic pain patterns Spine 2003 28 161 166 10.1097/00007632-200301150-00012 12544933

[B7] Efron B Tibshirani R Bootstrap methods for standard errors, confidence intervals, and other measures of statistical accuracy Statistical Science 1986 1 54 75 10.1214/ss/1177013815

[B8] Efron B Tibshirani R Statistical data analysis in the computer age Science 1991 253 390 395 10.1126/science.253.5018.390 17746394

[B9] Efron B Tibshirani R An introduction to the bootstrap 1993 New York: Chapman & Hall 1 436

[B10] Henderson AR The bootstrap: A technique for data-driven statistics. Using computer-intensive analyses to explore experimental data Clin Chim Acta 2005 359 1 26 10.1016/j.cccn.2005.04.002 15936746

[B11] Hays RD Kallich JD Mapes DL Coons SJ Carter WB Development of the kidney disease quality of life (KDQOL) instrument Qual Life Res 1994 3 5 329 338 10.1007/BF00451725 7841967

[B12] Ware JE Jr Kosinski M Keller SD A 12-item short-form health survey: Construction of scales and preliminary tests of reliability and validity Medical Care 1996 34 220 233 10.1097/00005650-199603000-00003 8628042

[B13] Lin P Ware JE Jr Meyer K Richardson M Bjorner JB Methods for psychometric and clinical evaluations of CAT-based measures of disease impact in chronic kidney disease (CKD) Value Health 2010 13 7 A244

[B14] Ware JE Jr Guyer R Harrington M Boulanger R Evaluation of a more comprehensive survey item bank for standardizing disease-specific impact comparisons across chronic conditions 2012 Budapest, Hungary: Invited presentation at International Society for Quality of Life Research (ISOQOL) conference

[B15] Evans RW Manninen DL Garrison LP Jr Hart LG Blagg CR Gutman RA Hull AR Lowrie EG The quality of life of patients with end-stage renal disease N Eng J Med 1985 312 9 553 559 10.1056/NEJM198502283120905 3918267

[B16] Evans RW Rader B Manninen DL The quality of life of hemodialysis recipients treated with recombinant human erythropoietin, Cooperative Multicenter EPO Clinical Trial Group J Am Med Assoc 1990 263 825 830 10.1001/jama.1990.03440060071035 2404150

[B17] Hansen RA Chin H Blalock S Joy MS Predialysis chronic kidney disease: evaluation of quality of life in clinic patients receiving comprehensive anemia care Res Social Adm Pharm 2009 5 2 143 153 10.1016/j.sapharm.2008.06.004 19524862 PMC2722114

[B18] Kerlinger FN Foundations of behavioral research 1973 New York: Holt, Rinehart, & Winston

[B19] Raczek AE Ware JE Jr Bjorner JB Gandek B Haley SM Aaronson NK Apolone G Bech P Brazier JE Bullinger M Sullivan M Comparison of Rasch and summated rating scales constructed from SF-36 physical functioning items in seven countries: Results from the IQOLA project J Clin Epidemiol 1998 51 1203 1214 10.1016/S0895-4356(98)00112-7 9817138

[B20] McHorney CA Haley SM Ware JE Jr Evaluation of the MOS SF-36 physical functioning scale (PF-40): II, Comparison of relative precision using Likert and Rasch scoring methods J Clin Epidemiol 1997 50 451 461 10.1016/S0895-4356(96)00424-6 9179104

[B21] Fitzpatrick R Norquist JM Dawson J Jenkinson C Rasch scoring of outcomes of total hip replacement J Clin Epidemiol 2003 56 1 68 74 10.1016/S0895-4356(02)00532-2 12589872

[B22] Norquist JM Fitzpatrick R Dawson J Jenkinson C Comparing alternative Rasch-based methods vs raw scores in measuring change in health Medical Care 2004 42 1 Suppl I25 I36 14707753 10.1097/01.mlr.0000103530.13056.88

[B23] Fitzpatrick R Norquist JM Jenkinson C Reeves BC Morris RW Murray DW Gregg PJ A comparison of Rasch with Likert scoring to discriminate between patients' evaluations of total hip replacement surgery Qual Life Res 2004 13 2 331 338 15085905 10.1023/B:QURE.0000018489.25151.e1

[B24] Hart DL Mioduski JE Stratford PW Simulated computerized adaptive tests for measuring functional status were efficient with good discriminant validity in patients with hip, knee, or foot/ankle impairments J Clin Epidemiol 2005 58 629 638 10.1016/j.jclinepi.2004.12.004 15878477

[B25] Hart DL Cook KF Mioduski JE Teal CR Crane PK Simulated computerized adaptive test for patients with shoulder impairments was efficient and produced valid measures of function J Clin Epidemiol 2006 59 290 298 10.1016/j.jclinepi.2005.08.006 16488360

[B26] Deng N Ware JE Jr Using bootstrap confidence interval to compare relative validity coefficient: an example with PRO measures of chronic kidney disease impact Value in Heal 2012 15 4 A159

[B27] Efron B Better bootstrap confidence intervals J Am Stat Assoc 1987 82 171 200 10.1080/01621459.1987.10478410

[B28] DiCiccio TJ Efron B Bootstrap confidence intervals Statistical Science 1996 11 189 228

[B29] R Development Core Team. R A language and environment for statistical computing 2011 Vienna, Austria: R Foundation for Statistical Computing URL http://www.R-project.org/

[B30] Canty A Ripley Bboot: Bootstrap R (S-Plus) functions. R package version 1.3-4 2012

[B31] Davison AC Hinkley DV Bootstrap methods and their applications 1997 Cambridge: Cambridge University Press

[B32] McHorney CA Ware JE Jr Raczek AE The MOS 36-item Short-Form health survey (SF-36): II. psychometric and clinical tests of validity in measuring physical and mental health constructs Medical Care 1993 31 3 247 263 10.1097/00005650-199303000-00006 8450681

[B33] Vickrey BG Hays RD Genovese BJ Myers LW Ellison GW Comparison of a generic to disease-targeted health-related quality-of-life measures for multiple sclerosis J Clin Epidemiol 1997 50 557 569 10.1016/S0895-4356(97)00001-2 9180648

[B34] Ware JE Jr Kosinski M Bjorner JB Bayliss MS Batenhorst A Dahlöf CG Tepper S Dowson A Applications of computerized adaptive testing (CAT) to the assessment of headache impact Qual Life Res 2003 12 8 935 952 10.1023/A:1026115230284 14651413

[B35] Carpenter J Bithell J Bootstrap confidence intervals: when, which, what? A practical guide for medical statisticians Statistics In Medicine 2000 19 1141 1164 10.1002/(SICI)1097-0258(20000515)19:9<1141::AID-SIM479>3.0.CO;2-F 10797513

[B36] Lindman HR Analysis of variance in complex experimental designs 1974 New York, NY: W. H. Freeman

[B37] Box GEP Some theorems on quadratic forms applied in the study of analysis of variance problems: II Effect on inequality of variance and correlation of errors in the two-way classification Annals of Mathematical Statistics 1954 25 484 498 10.1214/aoms/1177728717

